# Evaluation of Growth in Children with Inflammatory Bowel Disease

**DOI:** 10.3390/children11091038

**Published:** 2024-08-25

**Authors:** Uğur Altaş, Deniz Ertem

**Affiliations:** 1 Department of Pediatrics, Faculty of Medicine, Marmara University, Istanbul 34899, Türkiye; 2 Department of Pediatrics, Division of Pediatric Gastroenterology Hepatology and Nutrition, Faculty of Medicine, Marmara University, Pendik, Istanbul 34899, Türkiye

**Keywords:** growth, pediatric inflammatory bowel disease, Crohn’s disease, ulcerative colitis

## Abstract

Objective: This study aimed to evaluate changes in growth parameters in children diagnosed with inflammatory bowel disease (IBD). Methods: The data of children with IBD between 2010 and 2018 were retrospectively reviewed. Anthropometric measurements (height, weight, and BMI [body mass index]), and clinical and laboratory data were evaluated at diagnosis and follow-up (1st and 2nd year). Patients’ growth was assessed by calculating weight-for-age, height-for-age, BMI-for-age, and growth velocity z-scores. Results: Thirty-six patients (46.2%) had Crohn’s disease (CD), and 42 (53.8%) had ulcerative colitis (UC). Weight-for-age, height-for-age, and BMI-for-age z-scores significantly increased over the follow-up period in the CD patients (*p* < 0.05). Growth velocity z-scores were also significantly higher in the second year compared to the first year in the CD patients (*p* < 0.001). Improvements in weight-for-age, height-for-age, and BMI-for-age z-scores were not significant over the two-year follow-up in the UC patients (*p* > 0.05). Growth velocity z-scores in the UC patients were higher in the second year compared to the first year, but this difference was not significant (*p* = 0.115). Conclusions: The growth parameters showed improvement after a two-year follow-up. Regular anthropometric measurements, along with clinical and laboratory markers, should be used to monitor treatment response, which can help achieve optimal growth in children with IBD.

## 1. Introduction

Inflammatory bowel disease (IBD), a chronic inflammatory disease of the gastrointestinal system, progresses with relapses and remissions. Inflammatory bowel disease is more common in young adults and adolescents [1]. There have been reports of an increase in the incidence of IBD in the pediatric population in recent years [2]. There are three different subtypes of IBD: ulcerative colitis (UC), Crohn’s disease (CD), and IBD-unclassified [3].

Growth is one of the most important indicators of health in children [4]. Growth retardation and malnutrition are the most important and serious complications of children with IBD [5,6]. It has been reported that significant growth retardation is observed in children and adolescents with IBD at the time of diagnosis and during follow-ups. In one study, growth retardation and delayed puberty were observed in approximately one-third of children with Crohn’s disease [5]. In another study evaluating anthropometric measurements of 783 newly diagnosed children with IBD, it was reported that the body mass index (BMI) was below the fifth percentile in 22–24% of the children with CD and 7–9% of the children with UC [7]. Data from longer-term follow-ups also indicate ongoing growth retardation in children. A retrospective study conducted in France analyzed 107 children with Crohn’s disease and found that 29% of the children did not reach their target adult height [8]. A recent study also reported that approximately half of the children with CD and UC were able to reach their target adult height [9].

Factors such as inadequate calorie intake, malabsorption, the effects of inflammatory cytokines, genetic and endocrine factors, and corticosteroid use play a role in the pathophysiology of growth retardation in pediatric IBD. The deficiency of vitamin D can be frequently seen in patients with IBD [10]. Since vitamin D plays a role in calcium absorption and bone mineralization, it has positive effects on bone development and growth in children with IBD [11]. Similarly, deficiencies in micronutrients such as iron, B12, and zinc can have negative effects on growth and puberty [6,12]. The management of growth retardation in children with IBD aims to control chronic inflammation associated with the disease, taking these factors into account [13].

Since growth retardation can be common in children with IBD, evaluating growth retardation in children with UC and CD will guide clinicians in managing the disease. In this context, this study aims to evaluate the growth and anthropometric measurements of children followed up with a diagnosis of IBD, and to examine factors that may be associated with growth.

## 2. Materials and Methods

### 2.1. Study Type and Population

**Figure 1:**
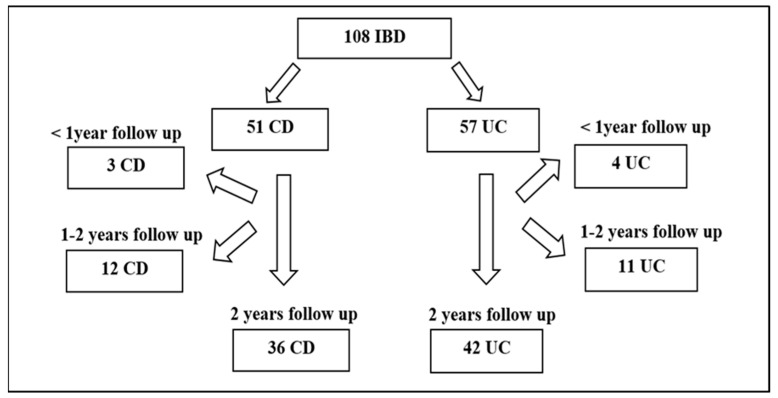
Distribution of the number of patients with IBD according to the duration of follow-up.

Within the scope of a retrospective cohort study, the data of 120 patients aged 5–18 years diagnosed with IBD between 2010 and 2018 and followed up in the Pediatric Gastroenterology, Hepatology, and Nutrition Department of a state university hospital were retrospectively reviewed. Two patients had malignancy, two had immunodeficiency, three had other chronic diseases besides inflammatory bowel disease, and five had missing data at the time of diagnosis, so twelve patients were not included in the study. There were remaining data available for 108 IBD patients, consisting of 51 CD and 57 UC cases. The effect of IBD on growth was investigated through 78 patients (36 with Crohn’s disease and 42 with ulcerative colitis) with complete 2-year follow-up data among the 108 IBD patients (Figure 1). No sample size was calculated for the study; instead, all the patients who met the eligibility criteria described above were included in the study.

### 2.2. Evaluation

Anthropometric measurements, and clinical and laboratory data at the time of diagnosis, as well as at the first and second year of follow-up, were evaluated along with demographic data. Among the laboratory values, hemoglobin, hematocrit, MCV, ferritin, CRP, ESR (Erythrocyte Sedimentation Rate), vitamin B12, vitamin D, and folate were examined. The presence of anemia in the patients was defined using the WHO hemoglobin standard values by age [14]. The growth of the patients was evaluated by calculating age-adjusted weight, height, BMI, and growth velocity z-scores at the time of diagnosis, and at the first and second year of follow-up. Body mass index was calculated using the formula “BMI = weight (kg)/height^2^”. The WHO AnthroPlus application prepared by the World Health Organization (WHO) was used for the z-score calculations for the weight-for-age, height-for-age, and BMI-for-age measurements [15]. The following formula was used for z-score calculation:

Z-score = (measured anthropometric value − mean value for age and sex)/standard deviation for age and sex.

To calculate the growth velocity z-score values, the AnthroCalc application, which uses the Kelly reference values from 2014, was used [16]. The following formula was used to calculate the growth velocity:

Growth velocity (cm/year) = (Final measurement (cm) − Initial measurement (cm))/Duration (months).

In the classification of disease activity, the Pediatric Crohn’s Disease Activity Index (PCDAI) and the Pediatric Ulcerative Colitis Activity Index (PUCAI) were used [17,18]. 

### 2.3. Statistical Analysis

The statistical analyses and data recording were performed using the SPSS 22.0 (Statistical Package for Social Sciences) program. Mean and standard deviation were used for the numeric values with normal distribution; median values and interquartile range (IQR) were used for the numeric values that were not normally distributed. Number (*n*) and percentages (%) were used for categoric data. The normal distribution of continuous variables was assessed visually (histograms and probability plots) and analytically (Kolmogorov–Smirnov/Shapiro–Wilk tests). For comparisons between the variables, the *t*-test was used for the normally distributed data in two-group comparisons, and the Mann–Whitney U test was used for the non-normally distributed data. Pearson correlation was used for the normally distributed continuous variables, while Spearman correlation was applied for the non-normally distributed continuous variables. Cochran’s Q test was utilized to evaluate the differences in proportions across three related groups for the categorical variables. The statistical significance of changes in anthropometric measurements at diagnosis and follow-up (1st and 2nd years) was assessed using the Friedman test and evaluated using the Bonferroni correction. The Wilcoxon test was used to measure the temporal change in growth velocity z-score from the 1st to the 2nd year. A *p* value of less than 0.05 was considered statistically significant.

### 2.4. Ethics

The study was approved by the Marmara University Faculty of Medicine Clinical Research Ethics Committee with protocol number 09.2018.416 on 1 June 2018.

## 3. Results

Of the patients with complete follow-up data for two years, 36 (46.2%) had CD and 42 (53.8%) had UC. Among the patients with Crohn’s disease, 22 (61.1%) were male and 14 (38.9%) were female; among those with ulcerative colitis, 18 (42.9%) were male and 24 (57.1%) were female. The mean age at diagnosis was 12.89 ± 3.34 years for Crohn’s disease and 11.51 ± 3.21 years for ulcerative colitis (*p* = 0.038). The rate of patients diagnosed under the age of 10 was 19.4% (*n* = 7) for Crohn’s disease and 35.7% (*n* = 15) for ulcerative colitis.

### 3.1. Findings about the Disease Clinic and Location

The majority of Crohn’s disease patients were diagnosed with ileocolonic involvement (L3) (*n* = 30, 83.3%). The numbers of patients with ileal (L1) and colonic (L2) involvement at diagnosis were two (5.6%) and four (11.1%), respectively. In 83.3% of the Crohn’s disease patients (*n* = 30), the disease behavior at diagnosis was inflammatory (B1) type. Strictures (B2), penetrating disease (B3), and a combination of B2 + B3 behavior were observed in two patients (5.6%) in each group.

Regarding the disease location at diagnosis in ulcerative colitis patients, 50% (*n* = 21) had E4 involvement, while the rates of E1, E2, and E3 involvement were 7.1% (*n* = 3), 28.6% (*n* = 12), and 14.3% (*n* = 6), respectively.

### 3.2. Findings Related to Disease Activity

Patients’ disease activities at diagnosis, 1st-, and 2nd-year follow-ups were evaluated based on the PCDAI and PUCAI scores. At diagnosis, 63.9% (*n* = 23) of the CD patients showed moderate to severe activity, while 88.9% (*n* = 32) were in remission at the end of the 2nd-year follow-up. The proportion of UC patients showing mild to moderate activity at diagnosis was 38.1% (*n* = 16) for both. However, in the second year of follow-up, 58.5% (*n* = 24) of the UC patients were in remission (Table 1).

### 3.3. Laboratory Findings

The laboratory results of the patients followed up with a diagnosis of Crohn’s disease at diagnosis, 1st year, and 2nd year are presented in Table 2. During the two-year follow-up period, a decrease was observed in inflammation markers CRP and ferritin, while an increase was detected in hemoglobin, hematocrit, MCV, vitamin D, and folic acid levels. Although the changes in ferritin and B12 vitamin levels were not statistically significant (*p* = 0.327 and *p* = 0.732), the positive changes in other laboratory values were statistically significant (*p* < 0.05) (Table 2). The rate of anemia detected at diagnosis in Crohn’s patients showed a statistically significant decrease in the 1st- and 2nd-year follow-ups (*p* < 0.001). While 76.5% of the patients (*n* = 26) had anemia at diagnosis, the rate of patients with anemia in the 1st year was 30.3% (*n* = 10), and in the 2nd year, it was 6.7% (*n* = 2) (Table 2).

During the 2-year follow-up of the UC patients, a decrease was observed in the inflammation marker CRP, while an increase was observed in hemoglobin, hematocrit, MCV, ferritin, vitamin D, and folic acid levels. Although the changes in ferritin and B12 and vitamin D levels were not statistically significant (*p* = 0.066, *p* = 0.717, and *p* = 0.053), the changes in the other laboratory values were statistically significant (*p* < 0.05) (Table 2).

The percentage of ulcerative colitis patients with anemia statistically significantly decreased in the 1st- and 2nd-year follow-ups compared to diagnosis (*p* < 0.001). In total, 76.7% of the ulcerative colitis patients (*n* = 23) had anemia at diagnosis, and this rate decreased to 33.3% (*n* = 10) in the 1st year and further to 20% in the 2nd year (*n* = 6) (Table 2).

### 3.4. Weight, Height, and BMI Values

**Figure 2:**
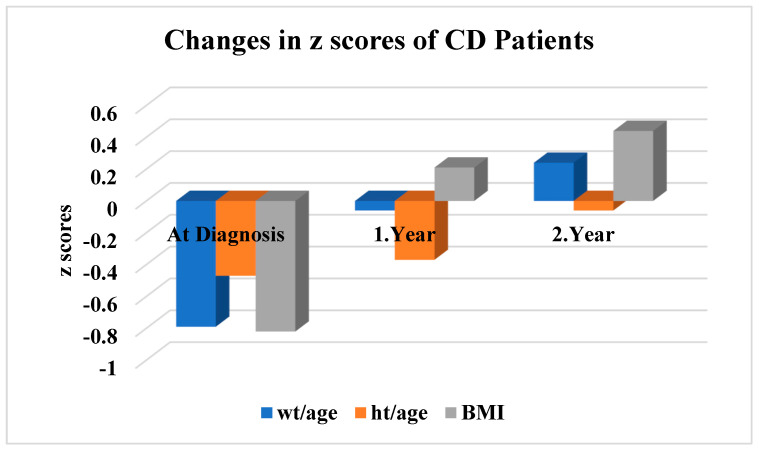
Anthropometric measurement z-scores of CD patients at diagnosis, 1st- and 2nd-year follow-up; weight z-score values by age: diagnosis vs. 1st year *p* < 0.001; 1st year vs. 2nd year *p* = 0.036; 2nd year vs. diagnosis *p* < 0.001; height z-score values by age: diagnosis vs. 1st year *p* = 0.252; 1st year vs. 2nd year *p* < 0.001; 2nd year vs. diagnosis *p* < 0.001; BMI z-score values by age: diagnosis vs. 1st year *p* < 0.001; 1st year vs. 2nd year *p* = 0.321; 2nd year vs. diagnosis *p* < 0.001.

The mean weight-for-age z-scores of the Crohn’s disease patients were −0.79 ± 1.31; −0.06 ± 1.13; and 0.24 ± 0.95 at diagnosis, 1st year, and 2nd year, respectively. The mean height-for-age z-scores were −0.47 ± 0.98; −0.37 ± 0.95; and −0.06 ± 0.89 at diagnosis, 1st year, and 2nd year, respectively. Mean BMI z-scores were calculated as −0.82 ± 1.45, 0.21 ± 1.10, and 0.44 ± 0.97, respectively. In the CD patients, weight-for-age, height-for-age, and BMI z-scores increased significantly during follow-up (*p* < 0.05) (Figure 2). 

**Figure 3:**
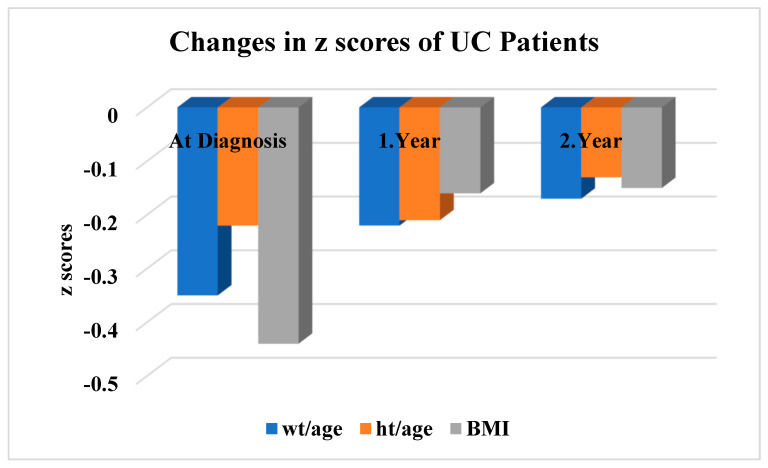
Anthropometric measurement z-scores of UC patients at diagnosis, 1st- and 2nd-year follow-up. *p* = 0.805 for weight z-score change by age, *p* = 0.521 for change in height z-score by age, and *p* = 0.462 for BMI z-score change by age.

The weight-for-age z-scores of the ulcerative colitis patients were −0.35 ± 0.93 at diagnosis, and −0.22 ± 0.75, and −0.20 ± 0.81 in years 1 and 2, respectively. The height-for-age z-scores were -0.22 ± 1.06, −0.21 ± 0.93, and −0.18 ± 0.85 at diagnosis, and in years 1 and 2, respectively. The BMI z-scores by age were −0.44 ± 1.37 at diagnosis, and −0.16 ± 1.02 and −0.15 ± 0.97 in years 1 and 2, respectively. Improvements in the weight-for-age, height-for-age, and BMI z-scores in the UC patients were not statistically significant at the two-year follow-up (*p* > 0.05) (Figure 3).

### 3.5. The Change in Growth Velocity during the 1st and 2nd Years of Follow-Up

**Figure 4:**
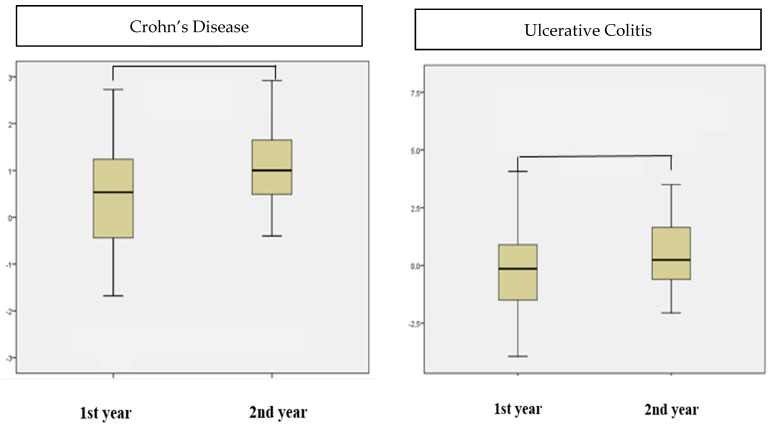
The growth velocity z-scores of the Crohn’s and UC patients in the 1st and 2nd years.

The 1st year growth velocity z-score of the Crohn’s patients was 0.32 ± 1.17, while it was 1.10 ± 0.83 in the 2nd year. When comparing the 1st and 2nd year growth velocities, the mean value of the 2nd year growth velocity z-score was significantly higher than that of the 1st year (*p* < 0.001) (Figure 4). The 1st year growth velocity z-score of the UC patients was −0.09 ± 1.86, and the 2nd year growth velocity z-score was 0.57 ± 1.59. There was no significant difference between the 1st and 2nd year growth velocity z-scores of the ulcerative colitis patients (*p* = 0.115) (Figure 4).

The effects of remission and steroid induction on growth velocities in IBD patients were examined. The mean growth velocity z-scores of both the CD and UC patients in remission were higher in the 1st and 2nd years compared to those not in remission, but statistical significance was only observed among the CD patients in the 1st year (*p* = 0.048). In the CD and UC patients, receiving steroid induction therapy at diagnosis did not have a significant effect on growth velocity in the 1st year (*p* > 0.05). In the CD patients, the mean growth velocity z-scores in the first and second years were higher in those diagnosed after the age of 10 years compared to those diagnosed before the age of 10 years, but statistical significance was not observed. Similarly, in the UC patients, the mean growth velocity z-score in the first year was higher in children diagnosed after the age of 10 years. However, in the second year, the growth velocity z-score was minimally higher in those diagnosed before the age of 10 years. Nevertheless, no significant relationship was observed between early age at diagnosis and growth velocity in the UC patients (Table 3).

The impact of laboratory values at diagnosis on growth velocity in the first year was evaluated in both the CD and UC patients. Similarly, the relationship between the laboratory values in the first year and growth velocity in the second year was assessed through correlation analysis. No significant effect of the laboratory values on growth velocity was found in the CD and UC patients (Table 4).

## 4. Discussion

Pediatric IBD patients face significant complications such as weight loss, malnutrition, and growth retardation, which can affect morbidity and quality of life in the long term. In patients where disease activity cannot be controlled during the peripubertal period, growth retardation can become an irreversible condition, and patients may not reach their target heights in adulthood [19]. In this context, this study examined the changes in growth parameters in children with CD and UC over a 2-year follow-up period.

In this study, when the anthropometric measurements of children with CD were evaluated, the weight z-score for age showed a statistically significant increase in the 1st and 2nd years of follow-up compared to the diagnosis. This improvement in weight may be attributed to both the control of disease activity and the appetite-stimulating effect of steroids used in the remission induction of Crohn’s patients. While the height z-score for the age of Crohn’s patients showed an increase in the 1st and 2nd years of follow-up compared to the diagnosis, the increase in the second year was more pronounced and statistically significant. This could be attributed to the negative effect of remission induction with steroids in the first year on bone growth. A study conducted on children with IBD showed a significant improvement in weight z-scores similar to this study over a 2-year follow-up period [20]. In this study, the BMI z-score of children with CD also showed an increase in the 1st and 2nd years of follow-up compared to the diagnosis, with a more pronounced increase in the first year, which was statistically significant. In a similar study from France, the growth of 261 children with CD was retrospectively evaluated over a period of 6 years, and like this study, a significant increase in weight and BMI z-scores for age was found, while no change was observed in height z-scores for age [21]. In this study, however, growth continued after disease remission in the Crohn’s patients, and there was even a significant increase in height in the 2nd year compared to the 1st year.

When the anthropometric measurements of the UC patients were evaluated in this study, a positive trend was observed in the weight z-score for age, height z-score for age, and BMI z-score for the age measurements in the 1st and 2nd years of follow-up after diagnosis, but this increase was not statistically significant. Since growth retardation is less common in UC patients compared to Crohn’s patients, there are fewer cohort studies evaluating growth in UC patients. Studies have mostly evaluated the growth parameters of UC patients at diagnosis [22]. In a study evaluating the height z-scores for the age of UC patients, there was no significant difference between the height z-scores for the age of UC pediatric patients and reference population measurements [23]. In a retrospective community-based study conducted in Japan between 2004 and 2011, which evaluated 2090 CD and 4600 UC patients under 20 years of age, both groups of patients were found to have significantly lower height z-scores at diagnosis compared to the reference values [6].

Inflammatory bowel disease requires the control of inflammation to achieve important goals such as improving quality of life and preventing growth impairment, especially in pediatric patients who have not yet completed linear growth. Clinical symptoms, acute-phase markers (CRP and ESR), PUCAI, and PCDAI are used to distinguish between active and remission states. In this study, the mean CRP values of the Crohn’s patients decreased in the 1st and 2nd years of follow-up compared to diagnosis. Consistent with this, the mean PCDAI scores of the patients in the 1st and 2nd years showed a statistically significant decrease compared to the diagnosis. In a study analyzing the national pediatric Crohn’s disease database (BELCRO) in Belgium, the proportion of Crohn’s patients showing moderate to severe activity decreased from 76% at diagnosis to 4% in the 1st and 2nd years. In the same study, while 5% of the patients were inactive at diagnosis, it was reported that 46% and 58% of the patients were in remission in the 1st and 2nd years, respectively [24]. In this study, the proportion of Crohn’s patients showing moderate to severe activity was 63.9% at diagnosis, but this proportion decreased to 2.8% in the 1st and 2nd years of treatment, indicating effective control of inflammation. The remission rate achieved in this study was 77.8% in the 1st year and increased to 88.9% in the 2nd year. The higher remission rates in this study compared to the BELCRO study may be due to the higher proportion of patients with moderate to severe activity in the BELCRO study (76% vs. 63.9% in this study).

In pediatric UC patients, as in Crohn’s patients, the goal is to control inflammation and prevent growth impairment. For this purpose, disease activity in UC is monitored using clinical findings, acute-phase markers, and PUCAI. In this study, the CRP and PUCAI values of the UC patients showed a significant decrease in the 1st and 2nd years of follow-up compared to diagnosis. More than 60% of the UC patients in this study were in moderate and severe disease activity at diagnosis, and accordingly, more than 61.9% of the patients had high CRP levels at diagnosis. In the 1st year, 47.6% of the UC patients were in remission, and in the 2nd year, 57.1% were in remission. In a study conducted in Scotland, remission was achieved in 57% of UC patients with moderate to severe disease activity in the 2nd year after treatment, similar to the results of this study [25].

In IBD patients, controlling the disease with treatment is known to have positive effects on growth. In this study, when growth velocities were evaluated, it was found that Crohn’s patients grew better in the 2nd year and the growth velocity z-score significantly increased. In a retrospective study conducted in Scotland, the 1st and 2nd year growth rate z-scores of Crohn’s patients were 0.0 [26]. The fact that the growth rates of Crohn’s disease patients in the 1st and 2nd years were found to be higher in this study compared to the other study may be related to disease activity. In this study, the CRP and ESR values were high in 24.2% and 39.2% of the patients in the 1st year. In the Scottish study, the CRP and ESR values were found to be high in 46.9% and 48.9% of the patients in the 1st year, respectively, indicating that inflammation was not under control [26]. The mean growth velocity z-score of UC patients in this study also increased in the 2nd year compared to the 1st year, but this increase was not statistically significant. In the literature, the number of studies including growth in the evaluation of treatment success in pediatric UC patients is quite limited. In a study conducted in Scotland, patients’ height measurement and Tanner stages were included in the evaluation of treatment response to biological agents. Remission was achieved in 57% of the UC patients who were all in moderate and severe disease activity in the 2nd year after treatment, but no significant improvement was observed in the growth rates of the patients [25].

### Limitations and Strengths

Potential confounding factors such as pubertal status, nutritional status, and adherence to therapy were not controlled for, which may influence growth outcomes. Conducting the study based on referrals to a single center also limits the generalizability of study results.

Despite these limitations, the study also has several strengths. The two-year follow-up period provides a substantial time frame to evaluate growth trends and identify significant changes in growth parameters among children with inflammatory bowel disease. The inclusion of both ulcerative colitis and Crohn’s disease patients allows for a comparative analysis, providing insights into the differential impact of these conditions on pediatric growth. This study contributes to the literature by evaluating the growth of children with both CD and UC after treatment, and by examining the factors that may be associated with growth. Additionally, the limited number of studies evaluating longitudinal growth in pediatric UC patients highlights areas where further research is needed. Furthermore, the findings offer valuable clinical information that can inform treatment strategies aimed at optimizing growth and development in pediatric patients with inflammatory bowel disease. Lastly, the use of detailed and standardized growth measurements enhances the reliability of the growth assessments.

## 5. Conclusions

In conclusion, this study observed that achieving remission with pharmacological treatment in pediatric patients with CD and UC led to improvements in growth parameters. This improvement was more pronounced in CD, where inflammation is more intense and systemic complications are more common compared to UC. Particularly, remission in the first year significantly increased growth velocity in the CD patients. In pediatric IBD patients, who are still growing and have a more severe disease course compared to adults, effective treatment to control inflammation should be initiated early, taking into account disease activity and extent. Regular anthropometric measurements, along with clinical and laboratory markers, should be used to monitor treatment response, which could help reduce the frequency of one of the most important complications, growth retardation, and achieve optimal growth. Crohn’s disease, in particular, may be accompanied by other chronic diseases. Patients with chronic conditions were not included in this study. These patients may be more severely affected by growth retardation. Therefore, in future studies, the growth and trace element deficiencies of IBD patients with comorbid conditions should also be evaluated.

## Figures and Tables

**Table 1:** The change in PCDAI and PUCAI scores of Crohn’s disease and ulcerative colitis patients at diagnosis, 1st-year, and 2nd-year follow-ups.

Crohn’s Disease Activity *	PCDAI at Diagnosis *n* (%)	1 Year PCDAI *n* (%)	2 Year PCDAI *n* (%)
Remission	-	28 (77.8)	32 (88.9)
Mild	13 (36.1)	7 (19.4)	3 (8.3)
Moderate-Severe	23 (63.9)	1 (2.8)	1 (2.8)
Disease Activity of Ulcerative Colitis **	PUCAI at diagnosis *n* (%)	1 year PUCAI *n* (%)	2 year PUCAI *n* (%)
Remission	-	20 (47.6)	24 (58.5)
Mild	16 (38.1)	20 (47.6)	11 (26.8)
Moderate	16 (38.1)	1 (2.4)	5 (12.2)
Severe	10 (23.8)	1 (2.4)	1 (2.4)

* Remission = 0–10 points; Mild = 11–30 points; Moderate-Severe = >30 points. ** Remission = 0–9 points; Mild = 10–34 points; Moderate = 35–64 points; Severe = ≥65 points.

**Table 2:** Laboratory values of Crohn’s disease and ulcerative colitis patients.

CD	At Diagnosis (Mean ± SD)	1 Year (Mean ± SD)	2 Year (Mean ± SD)	*p* Value
Hematocrit (%)	33.58 ± 4.67	37.07 ± 4.26	37.61 ± 4.05	<0.001
Hemoglobin (g/dL)	11.19 ± 1.51	12.26 ± 1.62	12.71 ± 1.49	<0.001
MCV (fL)	73.40 ± 7.32	79.39 ± 7.08	80.99 ± 6.91	<0.001
Ferritin (ng/mL)	63.93 ± 67.07	42.16 ± 35.48	19.4 (11.1–40.0) *	0.327
CRP (mg/L)	25.0 (8.0–54.0) *	6.4 (3.0–11.0) *	3.2 (3.0–14.0) *	<0.001
Vitamin B12 (pg/mL)	321.06 ± 201.68	286.0 (213.0–327.0) *	315.12 ± 119.37	0.732
Vitamin D (ng/mL)	13.22 ± 7.34	21.41 ± 8.83	21.22 ± 10.59	0.029
Folic acid (ng/mL)	5.52 ± 3.32	10.93 ± 6.16	6.9 (5.2–9.5) *	0.004
Presence of anemia (*n*, %)	26 (76.5)	10 (30.3)	2 (6.7)	<0.001
CRP positivity (>3 mg/L) (*n*, %)	25 (75.7)	8 (24.2)	6 (18.2)	0.056
ESR positivity (>20 mm/s) (*n*, %)	21 (63.6)	13 (39.2)	5 (15.2)	0.121
UC	At Diagnosis (Mean ± SD)	1 year (Mean ± SD)	2 year (Mean ± SD)	*p* value
Hematocrit (%)	32.90 ± 7.03	36.97 ± 5.23	37.17 ± 5.45	<0.001
Hemoglobin (g/dL)	10.98 ± 1.95	12.39 ± 2.07	12.37 ± 1.95	<0.001
MCV (fL)	73.65 ± 7.82	81.62 ± 6.20	81.84 ± 9.87	0.002
Ferritin (ng/mL)	8.9 (5.8–20.0) *	15.0 (4.6–27.0) *	16.0 (8.0–35.0) *	0.066
CRP (mg/L)	8.6 (3.0–17.0) *	3.0 (3.0–5.1) *	3.0 (3.0–3.1) *	0.001
Vitamin B12 (pg/mL)	383.07 ± 243.01	378.40 ± 214.42	451.22 ± 221.15	0.717
Vitamin D (ng/mL)	18.45 ± 15.60	21.28 ± 9.54	23.54 ± 14.26	0.053
Folic acid (ng/mL)	6.4 (2.7–23.0) *	11.24 ± 5.78	10.41 ± 6.14	0.001
Presence of anemia (*n*, %)	23 (76.7)	10 (33.3)	6 (20.0)	<0.001
CRP positivity (>3 mg/L) (*n*, %)	24 (61.5)	8 (22.8)	7 (20.4)	0.059
ESR positivity (>20 mm/s) (*n*, %)	18 (51.4)	10 (30.3)	4 (15.3)	0.225

* the data are presented as median (IQR) values due to non-normal distribution.

**Table 3:** The effects of remission status, age at diagnosis, and steroid induction on growth velocities in the IBD patients.

Crohn’s Disease			Ulcerative Colitis		
	**1st year growth velocity z-score**	***p* value**		**1st year growth velocity z-score**	*p* value
**Remission at 1 year**	Mean ± SD	0.048	**Remission at 1 year**	Mean ± SD	0.268
Yes (*n* = 28)	0.50 ± 1.07		Yes (*n* = 20)	0.15 ± 1.48	
No (*n* = 8)	−0.43 ± 1.26		No (*n* = 22)	−0.32 ± 2.16	
**Induction with steroids at diagnosis**	Mean ± SD	*p* value	**Induction with steroids at diagnosis**	Mean ± SD	*p* value
Yes (*n* = 25)	0.36 ± 1.10	0.986	Yes (*n* = 32)	−0.13 ± 1.89	0.790
No (*n* = 11)	0.23 ± 1.35		No (*n* = 10)	0.00 ± 1.87	
**Diagnosis < 10 years of age**	Mean ± SD	*p* value	**Diagnosis < 10 years of age**	Mean ± SD	*p* value
Yes (*n* = 7)	−0.21 ± 1.17	0.187	Yes (*n* = 15)	−0.37 ± 2.28	0.365
No (*n* = 29)	0.45 ± 1.15		No (*n* = 27)	0.05 ± 1.62	
	**2nd year growth velocity z-score**	*p* value		**2nd year growth velocity z-score**	*p* value
**Remission at 2 year**	Mean ± SD	0.597	**Remission at 2 year**	Mean ± SD	0.569
Yes (*n* = 32)	0.50 ± 1.07		Yes (*n* = 24)	0.89 ± 1.85	
No (*n* = 4)	−0.43 ± 1.26		No (*n* = 18)	0.33 ± 1.33	
**Diagnosis < 10 years of age**	Mean ± SD	*p* value	**Diagnosis < 10 years of age**	Mean ± SD	*p* value
Yes (*n* = 7)	1.08 ± 1.11	0.749	Yes (*n* = 15)	0.64 ± 2.19	0.431
No (*n* = 29)	1.11 ± 0.83		No (*n* = 27)	0.53 ± 1.18	

**Table 4:** The effects of the laboratory values on growth velocities in the IBD patients.

Crohn’s Disease			Ulcerative Colitis		
	**1st year growth velocity z-score**			**1st year growth velocity z-score**	
**Values at diagnosis**	r	*p*	**Values at diagnosis**	r	*p*
Hemoglobin (g/dL)	0.077	0.667	Hemoglobin (g/dL)	0.176	0.373
Ferritin (ng/mL)	−0.257	0.149	Ferritin (ng/mL)	−0.011	0.949
CRP (mg/L)	0.064	0.717	CRP (mg/L)	0.060	0.717
Vitamin B12 (pg/mL)	0.030	0.870	Vitamin B12 (pg/mL)	0.154	0.378
Vitamin D (ng/mL)	0.009	0.966	Vitamin D (ng/mL)	0.279	0.168
	**2nd year growth velocity z-score**			**2nd year growth velocity z-score**	
**Values at 1st year**	r	*p*	**Values at 1st year**	r	*p*
Hemoglobin (g/dL)	−0.241	0.208	Hemoglobin (g/dL)	−0.110	0.569
Ferritin (ng/mL)	0.061	0.776	Ferritin (ng/mL)	−0.180	0.349
CRP (mg/L)	−0.241	0.246	CRP (mg/L)	0.169	0.333
Vitamin B12 (pg/mL)	−0.096	0.703	Vitamin B12 (pg/mL)	−0.098	0.729
Vitamin D (ng/mL)	−0.142	0.550	Vitamin D (ng/mL)	−0.288	0.317

## Data Availability

The original contributions presented in the study are included in the article, further inquiries can be directed to the corresponding author.

## References

[1] Rabizadeh S., Dubinsky M. (2013). Update in pediatric inflammatory bowel disease. Rheum. Dis. Clin. North Am..

[2] Eszter Müller K., Laszlo Lakatos P., Papp M., Veres G. (2014). Incidence and paris classification of pediatric inflammatory bowel disease. Gastroenterol. Res. Pract..

[3] Loddo I., Romano C. (2015). Inflammatory bowel disease: Genetics, epigenetics, and pathogenesis. Front. Immunol..

[4] Amaro F., Chiarelli F. (2020). Growth and puberty in children with inflammatory bowel diseases. Biomedicines.

[5] Shamir R., Phillip M., Levine A. (2007). Growth retardation in pediatric Crohn’s disease: Pathogenesis and interventions. Inflamm. Bowel Dis..

[6] Ishige T. (2019). Growth failure in pediatric onset inflammatory bowel disease: Mechanisms, epidemiology, and management. Transl. Pediatr..

[7] Kugathasan S., Nebel J., Skelton J.A., Markowitz J., Keljo D., Rosh J., LeLeiko N., Mack D., Griffiths A., Bousvaros A. (2007). Body mass index in children with newly diagnosed inflammatory bowel disease: Observations from two multicenter North American inception cohorts. J. Pediatr..

[8] Ley D., Duhamel A., Behal H., Vasseur F., Sarter H., Michaud L., Gower-Rousseau C., Turck D. (2016). Growth pattern in pediatric Crohn disease is related to inflammatory status. J. Pediatr. Gastroenterol. Nutr..

[9] Choi S.Y., Choi S., Choe B.H., Park J.H., Choi K.H., Lee H.J., Park J.S., Seo J.H., Kim J.Y., Jang H.J. (2024). Factors associated with reaching mid-parental height in patients diagnosed with inflammatory bowel disease in childhood and adolescent period. Gut Liver.

[10] Pivac I., Jelicic Kadic A., Despot R., Zitko V., Tudor D., Runjic E., Markic J. (2023). Characteristics of the Inflammatory Bowel Disease in Children: A Croatian Single-Centre Retrospective Study. Children.

[11] Conklin L.S., Oliva-Hemker M. (2010). Nutritional considerations in pediatric inflammatory bowel disease. Expert. Rev. Gastroenterol. Hepatol..

[12] Bellini T., Bustaffa M., Tubino B., Giordano B., Formigoni C., Fueri E., Casabona F., Vanorio B., Pastorino A., Herzum A. (2024). Acquired and Inherited Zinc Deficiency-Related Diseases in Children: A Case Series and a Narrative Review. Pediatr. Rep..

[13] Sanderson I.R. (2014). Growth problems in children with IBD. Nat. Rev. Gastroenterol. Hepatol..

[14] Geneva S. (2011). Haemoglobin Concentrations for the Diagnosis of Anaemia and Assessment of Severity. Vitamin and Mineral Nutrition Information System. Document Reference WHO. NMH/NHD/MNM/11.1. https://www.who.int/publications/i/item/WHO-NMH-NHD-MNM-11.1.

[15] WHO 2009 Growth Reference Data. http://www.who.int/growthref/tools/en/.

[16] Kelly A., Winer K.K., Kalkwarf H., Oberfield S.E., Lappe J., Gilsanz V., Zemel B.S. (2014). Age-based reference ranges for annual height velocity in US children. J. Clin. Endocrinol. Metab..

[17] Hyams J.S., Ferry G.D., Mandel F.S., Gryboski J.D., Kibort P.M., Kirschner B.S., Griffiths A.M., Katz A.J., Grand R.J., Boyle J.T. (1991). Development and validation of a pediatric Crohn’s disease activity index. J. Pediatr. Gastroenterol. Nutr..

[18] Turner D., Otley A.R., Mack D., Hyams J., De Bruijne J., Uusoue K., Walters T.D., Zachos M., Mamula P., Beaton D.E. (2007). Development, validation, and evaluation of a pediatric ulcerative colitis activity index: A prospective multicenter study. Gastroenterology.

[19] Wine E., Reif S.S., Leshinsky-Silver E., Weiss B., Shaoul R.R., Shamir R., Wasserman D., Lerner A., Boaz M., Levine A. (2004). Pediatric Crohn’s disease and growth retardation: The role of genotype, phenotype, and disease severity. Pediatrics.

[20] Jin H.Y., Lim J.S., Lee Y., Choi Y., Oh S.H., Kim K.M., Yoo H.W., Choi J.H. (2021). Growth, puberty, and bone health in children and adolescents with inflammatory bowel disease. BMC Pediatr..

[21] Griffiths A.M. (2004). Specificities of inflammatory bowel disease in childhood. Best. Pract. Res. Clin. Gastroenterol..

[22] Mason A., Malik S., McMillan M., McNeilly J.D., Bishop J., McGrogan P., Russell R.K., Ahmed S.F. (2015). A prospective longitudinal study of growth and pubertal progress in adolescents with inflammatory bowel disease. Horm. Res. Paediatr..

[23] Mason A., Malik S., Russell R., Bishop J., McGrogan P., Ahmed S. (2011). Impact of inflammatory bowel disease on pubertal growth. Horm. Res. Paediatr..

[24] De Greef E., Hoffman I., Smets F., Van Biervliet S., Bontems P., Hauser B., Paquot I., Alliet P., Arts W., Dewit O. (2016). Pediatric Crohn disease: Disease activity and growth in the BELCRO cohort after 3 years follow-up. J. Pediatr. Gastroenterol. Nutr..

[25] Cameron F.L., Altowati M.A., Rogers P., McGrogan P., Anderson N., Bisset W.M., Ahmed S.F., Wilson D.C., Russell R.K. (2017). Disease status and pubertal stage predict improved growth in antitumor necrosis factor therapy for pediatric inflammatory bowel disease. J. Pediatr. Gastroenterol. Nutr..

[26] Malik S., Mason A., Bakhshi A., Young D., Bishop J., Garrick V., McGrogan P., Russell R.K., Ahmed S.F. (2012). Growth in children receiving contemporary disease-specific therapy for Crohn’s disease. Arch. Dis. Child..

